# Preventing and Treating Infection in Reverse Total Shoulder Arthroplasty

**DOI:** 10.1007/s12178-023-09843-1

**Published:** 2023-05-25

**Authors:** Alexander R. Markes, Joseph Bigham, C. Benjamin Ma, Jaicharan J. Iyengar, Brian T. Feeley

**Affiliations:** 1 Department of Orthopaedic Surgery, University of California-San Francisco, 1500 Owens Street, San Francisco, CA 94158 USA; 2 Department of Orthopaedic Surgery, University of Washington, Seattle, WA USA; 3 Stockton Shoulder Institute at Alpine Orthopaedics, Stockton, CA USA

**Keywords:** Prosthetic joint infection, Reverse total shoulder arthroplasty, Anatomic total shoulder arthroplasty, Prevention, Management, Diagnosis

## Abstract

**Purpose of Review:**

Periprosthetic infection after shoulder arthroplasty is relatively uncommon though associated with severe long-term morbidity when encountered. The purpose of the review is to summarize the recent literature regarding the definition, clinical evaluation, prevention, and management of prosthetic joint infection after reverse shoulder arthroplasty.

**Recent Findings:**

The landmark report generated at the 2018 International Consensus Meeting on Musculoskeletal Infection has provided a framework for diagnosis, prevention, and management of periprosthetic infections after shoulder arthroplasty. Shoulder specific literature with validated interventions to reduce prosthetic joint infection is limited; however existing literature from retrospective studies and from total hip and knee arthroplasty allows us to make relative guidelines. One and two-stage revisions seem to demonstrate similar outcomes; however, no controlled comparative studies exist limiting the ability to make definitive recommendations between the two options.

**Summary:**

We report on recent literature regarding the current diagnostic, preventative, and treatment options for periprosthetic infection after shoulder arthroplasty. Much of the literature does not distinguish between anatomic and reverse shoulder arthroplasty, and further high-level shoulder specific studies are needed to answer questions generated from this review.

## Introduction

Reverse total shoulder arthroplasty (TSA) is a common procedure for rotator cuff arthropathy, but indications have expanded in recent years [1]. These indications include but are not limited to proximal humerus fractures, revision arthroplasty, and glenohumeral arthritis [2]. With expanding indications frequently comes an expanding risk profile with complication rates after reverse TSA ranging from 7 to 25% in the reported literature. [2–7]. Of those complications, prosthetic joint infection (PJI) has an estimated incidence of 3–8% with median hospitalization cost of over 100% of that of the index procedure [2–11].

Despite the recent guidelines establishing a framework for management of PJI after total hip and knee arthroplasty, the diagnosis, prevention, and management of PJI after shoulder arthroplasty is less well-defined. The aim of this paper is to provide an in-depth review of literature published within the last 5 years, detailing evidence-based practices for clinical evaluation, prevention, and management of prosthetic joint infection after reverse total shoulder arthroplasty.

## Definition

In 2018, the International Consensus Meeting on Musculoskeletal Infection (ICM) met to establish a set of guidelines for the diagnosis, prevention, and management of periprosthetic joint infections after shoulder arthroplasty [12–15]. These diagnostic criteria were similar to those developed by the Musculoskeletal Infection Society (MSIS) in 2011 on diagnosis of PJI in the hip and knee [16]. The 2018 ICM defined four separate categories within the diagnosis of prosthetic shoulder infection: definite infection, probable infection, possible infection, and unlikely infection, as seen in Table 1. A definite infection is defined by presence by at least one of the following: (1) presence of a sinus tract, (2) gross intra-articular pus, or (3) two positive tissue cultures with phenotypically identical virulent organisms. In addition, a set of minor criteria seen in Table 2 are established with weighted scores as listed for definition of probable infection, possible infection, or unlikely infection. A total score of 6 or greater with an identified organism indicates probable infection. A score of 6 or greater without an identified organism indicates possible infection. A score of fewer than 6 with either a single positive culture with a virulent organism or a score of fewer than 6 with two positive cultures with a low-virulence organism indicates possible infection. A score of fewer than 6 with negative culture or a score of fewer than 6 only single positive culture with a lower-virulence organism indicates unlikely infection [12].

**Table 1:** Definition of prosthetic joint infection after shoulder arthroplasty (from Garrigues et al. [12])

Category	Definition
Definite infection	Presence of a sinus tract from skin surface to the prosthesis OR Gross intra-articular pus OR Two positive tissue cultures with phenotypically identical virulent organisms
Probable infection	Minor Criteria score ≥ 6 with an identified organism
Possible infection	Minor Criteria score ≥ 6 without an identified organism OR Minor Criteria score < 6 with 1 positive culture with virulent organism OR Minor Criteria score < 6 with 2 positive cultures with low-virulence organism
Unlikely infection	Minor Criteria score < 6 with negative cultures OR Minor Criteria score < 6 with only 1 positive culture with low-virulence organism

**Table 2:** Minor criteria for diagnosis of probable, possible, or unlikely infection after shoulder arthroplasty (from Garrigues et al.[12])

Minor Criteria	Weight
Unexpected wound drainage	4
Single positive tissue culture with virulent organism	3
Single positive tissue culture with low-virulent organism	1
Second positive tissue culture (identical low-virulence organism)	3
Humeral loosening	3
Positive frozen section (5 PMNs in ≥ 5 high-power fields	3
Positive preoperative aspirate culture (low or high virulence)	3
Elevated synovial neutrophil percentage (> 80%)*	2
Elevated synovial WBC count (> 3000 cells/µL)*	2
Elevated ESR (> 30 mm/h)*	2
Elevated CRP level (> 10 mg/L)	2
Elevated synovial ⍺-defensin	2
Cloudy fluid	2

*PMN* polymorphonuclear leukocyte, *WBC* white blood cell, *ESR* erythrocyte sedimentation rate, *CRP* C-reactive protein

*Beyond 6 weeks from recent surgery

## Patient Risk Factors

There have been numerous studies that suggest that gender plays a role in susceptibility to infection [8, 9, 17, 18]. Men have been found to have a significantly higher colonization of *Cutibacterium acnes* (*C. acnes*), formerly known as *Propionibacterium acnes*, around the shoulder joint with one study reporting a 81.6% *C. acnes* superficial colonization rate amongst males [19]. Patients younger than age 65 have been found to have a higher risk of PJI after Reverse TSA [9, 17, 20, 21]. The role of BMI as a risk factor for PJI is still unknown. Work done by Richards et al. found no association between BMI and shoulder PJI [18]. Though prior studies have shown that obesity has no impact on postoperative complications, Cogan et al. recently demonstrated in a large cross-sectional analysis of over 100,000 patients that patients with a higher BMI had higher odds of readmission, deep vein thrombosis/pulmonary embolism, superficial infection, and prosthetic joint infection [22, 23]. There have been mixed results on the impact of comorbidities on shoulder PJI. Numerous studies have reported there being no link between diabetes mellitus and PJI [9, 24, 25]. Cancienne et al. reported that wound complications increase as hemoglobin A1c reaches an inflection point of 8.0 mg/dL [26]. Revisional surgery after primary reverse TSA has been found to increase the risk of PJI [27]. Nezwek et al. reported a 6% infection rate with patients who had revision surgery versus a 2% infection rate with those patients who had no revision [27]. Prior ipsilateral shoulder surgery has also been shown to be a risk factor of subsequent PJI after reverse TSA [28, 29]. Gates et al. demonstrated a 45% positive intra-operative culture rate in patients with no clinical signs of infection and a history of prior ipsilateral shoulder surgery undergoing primary shoulder arthroplasty [28]. While the relevance of the cultures may be debated, Florschütz et al. demonstrated an increased risk (relative risk = 4.8) of shoulder PJI in patients with a history of prior non-arthroplasty shoulder surgery, compared to those with no prior ipsilateral shoulder surgery [29].

## History and Physical Examination

**Fig. 1:**
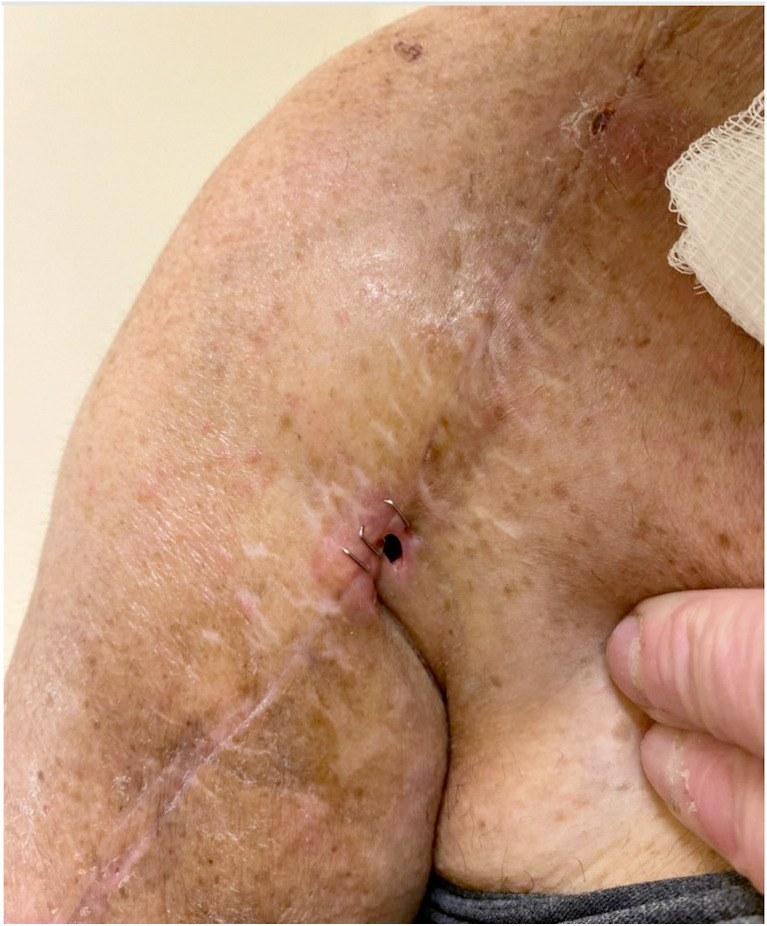
Clinical photo of draining sinus tract after shoulder arthroplasty

The history and physical examination for PJI of the shoulder has a wide range of clinical presentations. The spectrum of signs/symptoms includes erythema, limited range of motion, pain at surgical site, edema, stiffness, wound drainage, draining sinus, and/or exudative drainage [13]. Though rarely present, a draining sinus tract is pathognomonic for PJI, as seen in Fig. 1.

## Radiographic Evaluation

**Fig. 2:**
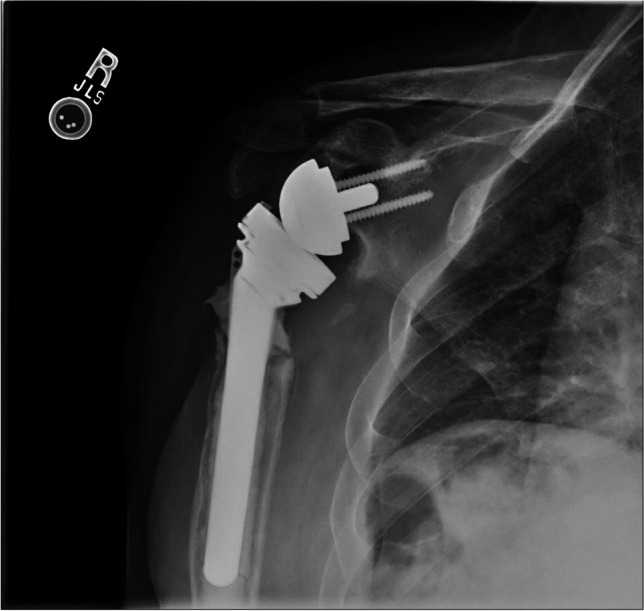
Radiographs of patient 3 years after reverse total shoulder arthroplasty with circumferential radiolucency between bone-cement interface, cortical erosion, and distal cement fracture concerning infection

The initial imaging modality in suspected prosthetic shoulder infection is plain radiographs (Fig. 2) to assess for any signs of implant loosening, bone erosion, osteolysis, and dislocation. Plain radiographs are not sensitive to infection for shoulder PJI, but it helps guide the surgeon to an accurate diagnosis when the clinical presentation aligns with typical radiographic findings. Computed tomography (CT) is essential for evaluating the shoulder before revisional surgery to assess hardware loosening and soft tissue edema or abscess formation. CT efficacy mirrors that of plain radiographs: not sensitive or specific to infection, although it can demonstrate the structural changes of bone and soft tissue often present in chronic shoulder PJI.

## Laboratory Evaluation

In addition to radiographs, a set of basic labs consisting of a complete white blood cell count (WBC) and inflammatory markers (ESR, CRP) is advised when PJI is suspected [13]. Although this is standard management, numerous studies suggest that these inflammatory markers are not sensitive or specific to shoulder PJI [13, 30]. A retrospective review analyzing the accuracy of ESR and CRP found an overall sensitivity of 16% and 42%, respectively, in the shoulder [31]. Increasingly, serum D-dimer levels are being used to aid in the diagnosis of PJI in hip and knee arthroplasty. Shahi et al., in a prospective study on 245 patients undergoing primary hip and knee arthroplasty, reported a D-dimer level of 850 ng/mL had a sensitivity of 89% and specificity of 93% for diagnosis of PJI [32]. Looking specifically at shoulder arthroplasty, Zmistowski et al. demonstrated elevated D-dimer in patients with definite or probable infections (median 661 ng/mL), compared with those with possible infections or those who were unlikely to have an infection (263 ng/mL). Taken in isolation, the diagnostic value is limited with a D-dimer level of 598 ng/mL, demonstrating 61% sensitivity and 74% specificity for diagnosing a definite or probable infection, according to the ICM definitions. [33] Further research is needed to evaluate the role of D-dimer in combination with other clinical, radiographic, and laboratory diagnostic markers of PJI.

## Synovial Fluid Analysis and Microbiology

Synovial fluid analysis may add valuable information in the initial workup for shoulder PJI. The 2018 ICM minor criteria for PJI is synovial WBC > 3000 cells/μL and neutrophil concentration > 80% [13]. Synovial fluid alpha-defensin is a lab value that has been widely studied in the diagnosis of PJI in the after total joint arthroplasty [34, 35]. Frangiamore et al. reported an alpha-defensin shoulder PJI sensitivity and specificity of 63% and 85%, respectively [34]. While this is lower than the reported sensitivity in hip and knee literature, it is still included in the minor criteria of the 2018 ICM definition of PJI [12, 36].

Numerous studies have demonstrated that *C. acnes* is the main culprit behind shoulder PJI. *C. acnes* is a gram positive rod found on skin flora and resides on sebum-rich pilosebaceous hair follicles [37]. Its indolent course and ability to make biofilms on prosthetic implants make it hard to detect, requiring cultures to be held for at least 14 days in an anaerobic medium [38]. Studies have implicated *C. acnes* in 28 to 79% all shoulder PJI [11, 12, 17, 37, 39, 40]. Specifically looking at revisions, Pottinger et al. reported that 70% were associated with *C. acnes* [20]. In a 2016 systematic review by Nelson et al., it was reported that *C. acnes* was implicated in 38.9% of all shoulder PJI, followed by *Staphylococcus aureus* at 14.8% and *Staphylococcus epidermidis* at 14.5% [41]. It is important to keep in mind that while helpful aspiration may not be negative though, this does not exclude infection of the shoulder.

## Prevention

### Optimizing Modifiable Risk Factors

As previously discussed, there are multiple risk factors associated with PJI, some of which can be modified to mitigate the risk of PJI. While corticosteroid injections are a common treatment used in the non-operative management of glenohumeral arthritis, use in the perioperative setting has been linked to increased risk of shoulder PJI. Werner et al. showed that patients who received an ipsilateral shoulder corticosteroid injection within 3 months prior to surgery had a 2 times increased risk of PJI. They found no increased risk of infection in patients that received an injection 3–12 months prior to arthroplasty [42].

Diabetes is also a known risk factor for poor wound healing after surgery, including shoulder arthroplasty. Querying a national database, Cancienne et al. reported a nearly 1.5 times odds of deep infection in patients with diabetes and using receiver operating characteristic analysis demonstrated a HbA1c of 8.0 mg/dL was a threshold to markedly increased risk of infection [26]. Despite this, there is still no validated study demonstrating use of a cutoff HbA1c level or evidence that delaying surgery until HbA1c is below a specific cutoff decreases risk of PJI.

Hatta et al. demonstrated that among patients who underwent reverse or anatomic TSA, current and former smokers had significantly higher risk of periprosthetic infection in comparison with non-smokers (hazard ratio [*HR*], 7.27 and 4.56, respectively) [43]. They defined former smokers as patient who had a documented history of tobacco use, in the form of cigarettes, cigars, or chewing tobacco during his or her lifetime but did not smoke within 1 month before surgery. Though they documented a lower hazard ratio of PJI in a former smoker using non-smokers as the reference, there was no significant difference when using multivariable analysis to directly compare being a former smoker to a current smoker. Overall, the causal effect of smoking cessation remains unclear in limiting PJI risk after TSA.

In terms of intra and post-operative modifiable risk factors, limiting blood loss may have some role in decreasing risk of PJI. Everhart et al. found perioperative blood transfusion increased risk of periprosthetic shoulder infection in a dose-dependent manner, with a relative risk of 1.86 times per unit of packed red blood cells [44]. Grier et al. similarly showed a two times greater odds of infection in patients who received a perioperative blood transfusion [45]. Prior studies have demonstrated treatment of pre-operative anemia before joint arthroplasty can decrease transfusion rates. Kotze et al. found using a protocol of erythropoietin, vitamin B12, or folate supplementation they was able to cut pre-operative anemia prevalence from 26 to 10% before elective total hip or knee arthroplasty [46]. It is not clear whether transfusion itself is associated with the development of shoulder PJI or if it is a proxy for poor surgical hemostasis and hematoma formation following reverse TSA that is the causative factor. Intraoperative use of antifibrinolytic agents, tranexamic acid (TXA), or ε-aminocaproic acid has also been shown to decrease transfusion rates in shoulder arthroplasty patients [47, 48]. Although TXA can pose a theoretical risk of thromboembolism in patients with prior history, it can be delivered topically which may be preferable in these patients and was found to be similarly effective to IV TXA in reducing blood loss and transfusion rates [48]. The 2018 ICM states there is no evidence for routine TXA administration to decrease PJI risk in the shoulder, Hong et al., however, reported lower odds (*OR* 0.49) of PJI after total hip or knee replacement with administration of TXA on the day of surgery [15, 49]. Further studies are needed to look at the direct efficacy of TXA protocols on shoulder PJI prevention.

### Topical Treatments and Skin Preparation

Literature on the use of different topical skin preparation treatments has demonstrated decreased bacterial load, though the efficacy of these treatments to decrease incidence of PJI is still unclear [50–55]. Surgical preparation with chlorhexidine and perioperative administration of cefazolin alone are not capable of eliminating the *C. acnes* bacterium on the skin because of its unique niche within pilosebaceous glands [55]. Addition of 5% benzoyl peroxide and 3% hydrogen peroxide has both been shown to decrease *C. acnes* bacterial burden on the skin without significant adverse reactions [50, 52, 55]. Despite this, the 2018 ICM states there is no evidence for or against the use of topical skin treatments to reduce of shoulder PJI; though they may present as a low cost, low-risk adjunct in the prevention of shoulder PJI [11, 15].

In terms of pre-operative scrubs and surgical preparation solution, the 2018 ICM does recommend chlorhexidine gluconate (CHG) showers or cleansing wipes with at least 2 applications decrease the incidence of positive skin culture findings prior to shoulder surgery [15]. Murray et al., in their randomized control trial, demonstrated a lower positive culture rate (66% vs 94%) after use of 2% chlorhexidine no-rinse cloths applied twice prior to shoulder surgery, compared with a control group that only used soap [56]. In addition, based on Level I evidence by Saltzman et al., the 2018 ICM recommended the use of 2% CHG and 70% isopropyl alcohol, such as ChloraPrep (Becton Dickinson, Franklin Lakes, NJ, USA), prior to shoulder arthroplasty [12, 57].

### Perioperative Antibiotic Prophylaxis

The 2018 ICM recommended that, for patients undergoing shoulder arthroplasty, a weight-based dose of IV cefazolin be administered within 30–60 min prior to incision; with redosing performed every 4 h, and, post-operative dosing not be extended beyond 24 h. For those with a serious β-lactam allergy, they recommended administration of Vancomycin, 15 mg/kg (max dose 2 g) within 1–2 h prior to incision with post-operative doses not to be given past 24 h [15]. For those with a personal history of MRSA infection or colonization, they recommended both vancomycin and cefazolin [15]. Use of clindamycin as an alternative in the setting severe β-lactam allergy has fallen out of favor after Yian et al. recently demonstrated a 3.5 times increased risk of infection in patients who underwent shoulder arthroplasty and received clindamycin alone, compared to those who received vancomycin or cefazolin [58].

There has been increasing use of vancomycin powder as an adjunct in the prevention of shoulder PJI, as it has been demonstrated in the total hip and knee literature to reduce incidence of PJI [59]. Miquel et al. recently demonstrated that administration of vancomycin powder to a bioartificial shoulder joint model shown to be biomimetic of shoulder PJI, completely eradicated *C. acnes* colonies analyzed 48 h after administration. Additionally, vancomycin powder had no discernible short-term impact on shoulder capsule cell morphology [60, 61]. Use of povidone-iodine solution lavage has also shown promising results of reduction in skin and soft tissue infections in the spine literature [15, 62, 63]. However, literature for its use in shoulder arthroplasty is deficient and its negative influence on osteoblast proliferation in vitro may limit its use in the setting of greater tuberosity fracture [64]. Furthermore, from a practical standpoint, the oblique orientation of the shoulder field due to modified beach chair positioning tends to preferentially promote lavage distribution to the inferior aspect of the wound. Consensus from the 2018 ICM is that both dilute povidone-iodine and/or vancomycin powder may have a role in patients considered at high risk of PJI, though more shoulder specific research is needed [15].

### Blue Light Therapy

In in vitro studies, *C. acnes* strains isolated from patients with shoulder PJI were found to be highly sensitive to blue light therapy in combination with specific photosensitizers [24]. The 2019 study by Grogan et al. was selected as the inaugural winner of the PJI research grant by the American Shoulder and Elbow Surgeons, though its clinical utilization is not yet widespread. The same research group is currently running a clinical trial on “Efficacy of blue light therapy at reducing bioburden of *C. acnes* at the deltopectoral interval” [65, 66]. Further investigation is clearly warranted but, if efficacious, it would represent an interesting novel treatment with a limited risk profile.

## Management

After diagnostic confirmation, various treatment options including non-operative treatment, one-stage revision, two-stage revision, and or multistage revision with open biopsies have been proposed for the management of shoulder PJI [40, 67, 68]. To help decide between which of these treatment options is the most appropriate one should consider multiple factors including duration of infection, patient overall health and risk profile, and desired functional goals.

### Operative Treatment Options

One-stage management includes irrigation and debridement with wide excision of infected tissue and either complete exchange of components or primary component retention. Intravenous antibiotic therapy is prescribed based on pre-operative or intra-operative culture sensitivities and can vary in duration in the literature [69]. Two-stage management includes complete implant removal and placement of an antibiotic spacer with a period of intravenous antibiotics that varies from 10 days to 3 months depending on patient response and responsible pathologic organism [14, 40, 67]. Reported techniques for the first stage of two-stage treatment widely vary in types of cement used, the amount of antibiotics in the cement, the use of prefabricated implants, or the use of intraoperatively handmade implants with or without the use of commercially designed spacer molds. Despite the varying techniques, there is little literature to suggest the superiority of a specific implant design or antibiotic cement regimen [70]. After clinical and laboratory markers suggest eradication of infection, the second stage is performed consisting of removal of the antibiotic spacer and implantation of revision components.

Tseng et al. has also described a three-stage revision protocol. In their protocol, the first stage consists of explant of components, thorough irrigation and debridement, placement of an antibiotic spacer, and a 6-week course of intravenous antibiotics. After completion of antibiotics, patients will undergo a 4-week antibiotic-free interval followed by an intermediate stage open biopsy to confirm eradication of infection. After cultures have been negative for at least 14 days, patients will return for removal of antibiotic spacer and implantation of revision components [71]. Zhang et al. similarly reported on such a technique, though also noted in his protocol if intermediate stage biopsy cultures were positive, then the patient would undergo another formal irrigation and debridement, exchange of the antibiotic spacer, and 6-week course of directed antibiotic therapy [68].

### Outcomes from One-stage vs Two-Stage Management

Two-stage management is standard of care for hip and knee PJI and generally considered the most reliable treatment for management of shoulder PJI [14, 40, 67, 72]. Despite this, based on the available evidence, single stage revision has been advocated to achieve similar infection control while minimizing perioperative risks and soft tissue deterioration associated with two stage procedures [69, 73]. In the 2018 ICM, a meta-analysis aggregated 161 cases (12 articles) of PJI, managed with single-stage and 325 cases (27 articles), and managed with 2-stage revision [14]. In their meta-analysis, they found a lower reinfection rate (5.6% vs 11.4%) and lower complication rate (12.7% vs 21.9%) after one-stage management, compared with two-stage management. Final reported functional outcome scores were also similar between groups (Constant-Murley score 49.1 for one-stage and 51.1 for two-exchange) [14].

These aggregated results do not take into timing of infection between acute, subacute, and chronic. This may add additional selection bias, particularly in the setting of more chronic and challenging revision cases where there is a preference towards two-stage procedures. Although there is inconsistent reporting of timing of infection, the majority of studies that report timing of infection classify acute as < 3 months, subacute as 3–12 months, and chronic as > 12 months [14]. Looking specifically at acute PJI, the 2018 ICM found 6 cases were treated with one-stage management, none of whom had reinfection at final follow-up [14]. With such few cases, the committee was unable to make concrete recommendations on management of acute PJI with one- vs two-stage revision.

Regarding management of subacute and chronic PJI, the 2018 ICM found four studies evaluating revision success rate for shoulder PJI with single-stage exchange between subacute or chronic presentation with a reinfection rate of 12.5% for chronic cases and 5.3% for subacute cases. They additionally found three studies specifically looking at success rate for two-stage exchange for sub-acute or chronic PJI with a reinfection rate of 6.3% for chronic cases and 29.4% for subacute cases [14]. They argue that selection bias may have a large role in the discrepancy with more severe infections being treated with two-stage revision [14]. Overall single stage management appears a promising and viable option for treatment of shoulder PJI; however, however there are no studies controlling for various risk factors such as pathogens, timing of infection, and diagnostic features. As such, the 2018 ICM was unable to make a strong recommendation for use of single-stage exchange in place of 2-stage exchange for shoulder PJI [14].

Multiple stage procedures with intermediate stage biopsy has been utilized by Zhang et al. given concerns for latent infections after standard 6-week antibiotic therapy. They demonstrated in their study looking at patients undergoing treatment with their multistage method for shoulder PJI; 22% of patients had evidence of persistent infection during their open biopsy procedure. All 18 patients treated with intermediate stage open biopsy in their study showed no signs of recurrent infection at 24 months after reimplantation [68]. Similarly, Tseng et al. looked at a matched cohort of 27 patients who underwent three-stage revision to RTSA for PJI, as described by Zhang et al., compared with 27 patients who underwent revision to RTSA for aseptic indications. They found no differences between infected and non-infected revisions in range of motion for forward flexion (121° ± 33° vs. 129° ± 30), abduction (117° ± 41° vs. 115° ± 36°), external rotation (29° ± 27° vs. 35° ± 21°), internal rotation (L4 vs. L2), VAS pain score (1.71 ± 1.76 vs. 1.33 ± 1.72), or ASES subjective score at the final follow-up (71.4 ± 22.7 vs. 74.3 ± 14.0)[71].

Overall surgeons must take multiple factors into account when determining treatment protocols including patient comorbidities, the risk of missing subclinical indolent infections, longer hospital stays with more aggressive treatment, and the risk of multiple operative interventions [40, 68].

### Single Stage with Implant Retention

Specifically looking at one-stage revision, some surgeons elect to retain the primary implants at time of surgery, though studies demonstrate low rates of overall eradication of infection in both the acute and chronic settings with this method [14]. In the acute setting, the 2018 ICM looked at 4 studies (38 shoulders) of patients treated with I&D and implant retention and found a 50% failure rate [14, 74–77]. They demonstrated similar findings in the case of subacute and chronic PJI with a 47% eradication rate [14]. Despite this, eradication of infection must be weighed with other risks of surgery. A French multicenter study looking at patients with PJI after reverse shoulder arthroplasty reported a 15% complication rate and 54% infection eradication rate after debridement, modular component exchange, and partial component retention. While the risk of residual infection was high, the complication rate was lower than that reported for resection (33%), 1-stage revision (20%), or 2-stage revision (36%) [78]. Overall, component retention should remain an option particularly in physiologically frail patients in whom more aggressive surgery incur significant perio-operatie risk, but clear informed consent between the surgeon and patient on the substantially higher rate of infection recurrence is vital.

### Unexpected Positive Culture

Unexpected positive cultures (UPC) at time of revision arthroplasty present a unique challenge with limited literature on outcomes to guide treatment recommendations. Hsu et al. reported on 55-revision shoulder arthroplasties without infection treated with 3 weeks of antibiotics, compared to those with > 2 UPCs treated with 6 months of antibiotics. They demonstrated no difference in pain or functional outcomes [79]. Padegimas also reported on 28 individuals with UPC after revision shoulder arthroplasty, compared to 89 individuals with negative cultures. Individuals were treated with 2 weeks of oral antibiotics followed by 6 weeks of additional antibiotics for those treated for infection. A higher percentage of patients with UPC underwent reoperation (20.2%) than those without (7.1%), but this difference was not statistically significant [80]. Overall, the lack of comparative data on outcomes and the significance of these cultures are still debated, making it difficult to conclusively determine optimal management. Moreover, the unclear clinical significance of UPC makes use of the 2018 ICM criteria a valuable tool in objectively placing the role of positive cultures in a broader context of PJI risk.

### Treatment of Low-Demand or High Perioperative Risk Individuals

Definitive treatment with irrigation and debridement, implant removal, and antibiotic spacer placement without plan for reimplantation of arthroplasty components is an option to be considered for select patients. Pelligrini et al. reported no recurrent infections with good pain relief and improvement in outcome scores in 19 patients with shoulder PJI treated definitively with antibiotic spacer [81]. Jawa et al. similarly looked 12 patients who underwent antibiotic spacer placement for shoulder PJI as definitive treatment after declining to proceed with the second stage and found a higher infection recurrence rate of 18% [82]. Though this technique is not without complications, McFarland et al. evaluated outcomes of patients who had antibiotic spacer placed after shoulder PJI and reported 18 complications in 14 patients including glenoid and humeral shaft erosion, spacer fracture or migration, and humerus fractures [83].

Alternatively, one can consider non-operative treatment with a course of intravenous antibiotic therapy followed by chronic oral suppressive therapy. Most of the literature of this technique comes from our understanding of its role in other arthroplasty procedures with the 2018 ICM only identifying 8 total shoulder cases treated with chronic suppressive therapy in their systematic review [14]. Additionally, most studies report on chronic suppressive therapy after initial surgical procedure such as evacuation of abscess or debridement. As such, the 2018 ICM was unable to give a recommendation for the type and duration of suppressive therapy in shoulder PJI but did acknowledge that it may have a role in select cases such as those who have high risk of perioperative complications with revision surgery [14].

## Conclusion

Prosthetic joint infection after shoulder arthroplasty is a low-risk complication, though it can lead to significant morbidity for those patients affected. The 2018 International Consensus Meeting on Musculoskeletal Infection established a framework for proper diagnosis and general management; through the efficacy of mitigation protocols, staged versus non-staged surgical treatment, and appropriate choice and duration of antibiotics can be evaluated. Current management strategies for diagnosis and management of shoulder PJI were largely extrapolated from hip and knee arthroplasty literature, although it is becoming increasingly clear that the microbial environment and soft tissue considerations for the shoulder joint are unique. Further shoulder-specific, high-level evidence is needed to better guide future recommendations in the management of prosthetic joint infections.
